# Class I HDAC Inhibitor Improves Synaptic Proteins and Repairs Cytoskeleton Through Regulating Synapse-Related Genes *In vitro* and *In vivo*

**DOI:** 10.3389/fnagi.2020.619866

**Published:** 2021-01-19

**Authors:** Ying Han, Le Chen, Yu Guo, Chunyang Wang, Chenghong Zhang, Li Kong, Haiying Ma

**Affiliations:** Department of Histology and Embryology, College of Basic Medical Sciences, Dalian Medical University, Dalian, China

**Keywords:** Alzheimer’s disease, β-amyloid, HDAC inhibitor, synaptic protein, cytoskeletal protein

## Abstract

β-amyloid (Aβ) is an important protein molecule in the pathology of Alzheimer’s disease (AD). Accumulation of Aβ leads to the loss of dendritic spines and synapses. These impairments can be ameliorated by histone deacetylase inhibitors (HDACI). However, the mechanisms of HDACIs underlying the effect on synapse are not fully understood. In this study, we examined the relationship between HDAC activity and synapse-related genes and proteins by the administration of a class I HDAC inhibitor, BG45, in the exogenous Aβ-treated cells and mice. Our studies showed that the treatment of HF-488-Aβ_1–42_ to SH-SY5Y cells first increased the expression of the postsynaptic dendritic protein (PSD), then decreased it after 36 h. BG45 can alleviate the reduction of the expression of PSD-95 as well as spinophilin and cytoskeletal protein induced by HF-488-Aβ^1–42^ aggregation in SH-SY5Y cells. Similar to the results *in vitro*, PSD-95 in the hippocampus was temporarily increased in the early days of intravenous injection HF-488-Aβ_1–40_ to the mice, followed by the decreased expression of PSD-95 on the 9th day. In further studies, for the mice treated with Aβ for 9 days, we found that BG45 decreased the expression of HDAC1 and 2, increased the expression of PSD-95, spinophilin, and synaptophysin (SYP). Our data also showed that BG45 upregulated levels of three synapse-related genes and proteins *GRIK2*, *SCN3B*, and *SYNPR*. These findings suggest that the exogenous Aβ may stimulate transiently the expression of PSD-95 at an early stage, but subsequently contribute to synaptic defects. HDAC1 and 2 are involved in synaptic defects, and BG45 may improve the expression of synaptic and cytoskeletal proteins and repair cytoskeletal damage by specifically inhibiting HDAC1 and 2, thereby modulating synapse-related genes. BG45 might be a potential therapeutic agent for the treatment of an early stage of Aβ-related neurodegenerative disease.

## Introduction

Alzheimer’s disease (AD) is an age-related, irreversible chronic neurodegenerative disease. Even though it is generally believed that the pathogenesis of AD is related to genetic factors, neurotransmitters, immune and environmental factors, et cetera (Bertram and Tanzi, 2008), the pathogenesis of AD is still unclear. Many researchers believed that the main reason for the failure to clinically treat AD is late intervention (Koppensteiner et al., 2016).

Many studies have focused on understanding the relationship between early pathology and AD pathogenesis (Selkoe, 2001; Julie and Laure Verret, 2010). AD is pathologically characterized by the accumulation of the β-amyloid peptide (Aβ) and intraneuronal neurofibrillary tangles (NFTs), which result in significant neuron loss and synapse dysfunction in areas of the brain responsible for cognition. Studies have shown that insoluble Aβ deposits have little to do with the development of AD. In contrast, soluble Aβ oligomers are closely related to AD, and they have stronger synaptic toxicity and neurotoxicity than Aβ plaques (Benilova et al., 2012). However, Roy et al. (2016) demonstrated that before the appearance of senile plaques and NFTs, pathological changes such as synaptic damage, decreased density of dendritic spines, and weakened synaptic connections between the entorhinal cortex and hippocampus have occurred in the brain (Freund et al., 2016). At the same time, other researchers found that although Aβ eventually causes inflammation and cell death, Aβ can act similar to antibacterial peptides early in killing common pathogenic microorganisms, and AD brain homogenate has stronger antibacterial activity than non-AD brain homogenate (Soscia et al., 2010; White et al., 2014). In any case, none of the clinical drugs against Aβ had satisfactory results. Therefore, rescuing synaptic damage before the appearance of Aβ oligomers may be very important in the prevention and treatment of AD.

Histone deacetylases (HDACs) are a class of proteases that play an important role in the structural modification of chromosomes and the regulation of gene expression (Eberharter and Becker, 2002). In humans, HDACs are categorized into four main classes: class I (HDACs 1, 2, 3, and 8), class II (HDACs 4, 5, 6, 7, 9, and 10), class III sirtuins (SIRT 1, 2, 3, 4, 5, 6, and 7), and class IV (HDAC 11; Citraro et al., 2017). HDAC inhibitors (HDACIs) rescue the impaired long-term potentiation, induce synapse formation, and increase hippocampal dendritic spine density (Rumbaugh et al., 2015). The expression of HDAC2 was significantly increased *in vivo* and *in vitro*, and it plays a role in modulating synaptic plasticity and long-lasting changes in neuronal circuits (Guan et al., 2009; Johannes et al., 2012). Overexpression of HDAC2 in neurons can also decrease the dendritic spine density, the number of synapses, and synaptic plasticity and memory (Guan et al., 2009). These results indicated that HDAC2 has a negative regulatory effect on memory formation and synaptic plasticity (Johannes et al., 2012).

In this study, human neuroblastoma cells SH-SY5Y and Kunming mice were treated with HF-488-Aβ^1–42^ and HF-488-Aβ_1–40_ monomer, respectively, for making AD models. We explored the correlation between HDAC1, 2, and early synaptic proteins and further investigated the effect of BG45, targeting of class I HDACs, *in vitro* and *in vivo*. The findings indicate that BG45, a class I-specific HDACI, significantly improves the expression of synaptic and cytoskeletal proteins and repairs cytoskeletal damage. The underlying mechanisms will be discussed. These results may provide theoretical support for early clinical intervention and treatment of AD.

## Materials and Methods

### Cell Culture, Differentiation, and Treatment

The human neuroblastoma cell line SH-SY5Y was cultured in DMEM media (Gibco, USA) supplemented with 10% fetal bovine serum (FBS; BI, USA), and 1% penicillin/streptomycin in a 5% CO_2_ humidified atmosphere at 37°C. Cell differentiation was induced by treatment with 10 μM all-trans retinoic acid (RA) for 7 days (Teppola et al., 2015).

Cells were treated with 200 nM HiLyte^TM^ Fluor 488-labeled β-Amyloid_(1–42)_ (HF-488-Aβ_1–42_, Anaspec Peptide, AS-60479-01) for different amounts of time for assessment by western blotting and immunofluorescence staining (Widenbrant et al., 2006). Cells were treated with 0, 10, 15, 20, or 25 μM BG45 (Selleck, 926259-99-6) for 24, 36, or 48 h for cell viability assays; cells in the control group were treated with vehicle (0.05% DMSO).

### CCK-8 Assay

Cell viability was measured in 96-well plates with CCK-8 assays. Briefly, after cells were treated with HF-488-Aβ_1–42_/BG45 or a vehicle for the indicated time, 100 μl of CCK-8 was added to the medium, and then cells were incubated at 37°C for 1 h. The absorbance at 450 nm was measured using a microplate reader (Thermo Fisher Scientific, Waltham, MA, USA). Cell viability is expressed as the ratio of the signal obtained from the treated group to the control group.

### Animals and Treatment

Kunming mice were provided by the Institute of Genome Engineered Animal Models for Human Disease, Dalian Medical University. All procedures were approved by the Institutional Animal Care and Use Committee of the Dalian Medical University in Dalian, China. Thirty-five mice were randomly divided into control group, and HF-488-Aβ_1–40_-treated group on different days (1, 3, 5, 6, 7, and 9 days) for screening out the suitable injection duration of Aβ. Fifteen mice were divided into control group, HF-488-Aβ_1–40_-treated group (HF-488-Aβ group) and BG45 administration group (HF-488-Aβ + BG45 group) for following experiments. The mice were all 8-week-old males. Intravenously injections were performed by a total volume of 100 μl of 0.5 μg HiLyte^TM^ Fluor 488-labeled β-Amyloid_(1–40)_ (HF-488-Aβ_1–40_, Anaspec Peptide, AS-60491-01) in sterile isotonic saline for 9 days in the HF-488-Aβ group and the HF-488-Aβ + BG45 group, vehicle injection in the control group. At the same time, 30 mg/kg BG45 was injected intraperitoneally in the HF-488-Aβ + BG45 treated group (Rumbaugh et al., 2015). After the neck was removed with sterile scissors, the cranium was cut from the back of the neck to the nose of mice and then the entire brain was carefully removed with forceps. The brain was placed on a plate containing PBS. Using a sterile scalpel, the cerebellum was removed and then cut down the midline of the brain, dividing it into two hemispheres. With sterile forceps, a section of the meninges around the hippocampus was gently pulled apart, revealing the hippocampus, which is actually a folding of the distal part of the cortex itself, lying just below the cerebral cortex and beginning at the distal end of the cerebral hemisphere and curving ventrally. Therefore, to isolate the hippocampus, it needs to be cut along the convex outer side (Seibenhener and Wooten, 2012). After the hippocampus was separated, sterile tissue forceps were used to gently lift the hippocampal tissue, and appropriate preservation methods were selected according to the experimental design.

### Western Blot Assay

Cells were lysed on ice with precooled lysis buffer. For animal samples, the stripped hippocampal tissue was cut up on ice and then added with precooled lysis buffer (100 g tissue plus 500 μl lysate). The homogenate was obtained by ultrasound 15 times. The homogenate was left at 4°C for 30 min. After centrifugation at 12,000 *g* for 15 min at 4°C, the total protein concentration of each experimental group was quantified using a BCA kit (Beyotime Biotechnology, China). Equivalent protein lysates (30 μg) were separated by 10% SDS–PAGE and blotted onto PVDF membranes (Millipore, USA); membranes were then blocked with 5% nonfat milk in Tris buffer at room temperature for 2 h. Western blotting was performed by incubation overnight at 4°C with the following antibodies: rabbit postsynaptic density protein 95 (PSD-95, 1:1,000, Abcam, ab18258), rabbit synaptophysin (SYP, 1:1,000, Abcam, ab32127), rabbit spinophilin (1:1,000, Cell Signaling Technology, 14136), rabbit tau (1:1,000, Cell Signaling Technology, 46687), rabbit p-tau (1:1,000, Cell Signaling Technology, 20194), rabbit HDAC1, rabbit HDAC2 (1:1,000, Cell Signaling Technology, 65816), mouse GAPDH (1:5,000, Proteintech, 60004-1) and rabbit β-actin (1:1,000, ABclone, AC026). After washing three times with 1× TBST, blots were incubated with a secondary antibody at room temperature for 1 h and were then washed again as before. Signals were visualized with an Amersham ECL Western Blotting Detection Kit (GE Healthcare Life Sciences) and were quantified using Quantitative One Image Analysis (BioRad, USA).

### Immunofluorescence Staining

Cells were seeded on 12-well slides (Solarbio, China) and treated as described above. The slides were washed with PBS, fixed with 4% PFA at room temperature for 20 min, washed three times with PBS, and permeabilized in a 0.5% Triton X-100 solution for 10 min. After washing with PBS, nonspecific antibody sites were blocked by incubating with 5% BSA at room temperature for 1 h. Slides were then incubated with one of the following primary antibodies overnight at 4°C: rabbit NeuN (1:200, Abcam, ab177487). After washing with PBS, the samples were incubated with one of the following secondary antibodies at room temperature for 2 h: Alexa Fluor-488 or Alexa Fluor-647 conjugated goat anti-rabbit (1:300, Vector Laboratories, USA). Then the samples were incubated with the nuclear dye DAPI at room temperature for 10 min. Cells were examined using a fluorescence microscope (Olympus, Japan), and the mean optical density was quantified using Image-pro plus 5.1 software.

For observing the distribution of HF-488-Aβ_1–40_ in the brains, mice in each group were anesthetized by intraperitoneal injection of 1% pentobarbital sodium solution (5 mg/100 g). The brains were harvested on ice into a 4% precooled paraformaldehyde fixation solution. The tissue was dehydrated in different concentrations of sucrose solution and embedded in OCT (SAKURA, Japan). The samples were cut into coronal frozen sections (slice thickness 10 μm). The slides were washed three times with PBS, incubated with the nuclear dye DAPI at 37°C for 15 min. After washing with PBS, the samples were observed using a fluorescence microscope.

### Labeling Cytoskeletal F-Actin

Cells were seeded on 12-well slides (Solarbio, China) and treated as described above. The slides were washed with PBS, fixed in a 4% PFA solution, and rinsed three times with PBS. The cells were permeabilized for 5 min in a 0.5% Triton X-100 solution. After washing with PBS, the cells were labeled with fluorescein phalloidin (1:200, Sigma–Aldrich) for 20 min at room temperature. After washing with PBS, the cells were stained with DAPI at room temperature for 10 min. The cells were viewed using a fluorescence microscope (Olympus, Japan), and the mean optical density was quantified using Image-pro plus 5.1 software.

### Real-Time Quantitative PCR (qPCR)

Total RNA from the hippocampus was extracted using Trizol reagent (Takara) and transcribed using a Reverse Transcription Kit (Applied Biosystems) according to the manufacturer’s instructions.

Primers for qPCR were used as follows:

*SCN3B*, F: 5′-ATGTGTCCAGGGAGTTTGAGT-3′

R: 5′-TTCGGCCTTAGAGACCTTTCT-3′

*SYNPR*, F: 5′-CAGCATTGACATAGCGTTTGC-3′

R: 5′-GGTTGTTTTCGCGGTACTTGT-3′

*GRIK2*, F: 5′-CAGCGTCGGCTCAAACATAAG-3′

R: 5′-GGTTTCTTTACCTGGCAACCTT-3′

*GAPDH*, F: 5′-TGTGATGGGTGTGAACCACGAGAA-3′;

R: 5′-GAGCCCTTCCACAATGCCAAAGTT-3′.

mRNA samples were mixed with primers and 2× TransStart Top Green qPCR SuperMix (Transgene) in a total volume of 20 μl. The thermal cycling conditions used in the protocol were 30 s at 94°C, followed by 45 cycles at 94°C for 5 s 60°C for 30 s and dissociation stage. Gene expression levels were analyzed relative to the level of the GAPDH gene transcript.

### Statistical Analysis

All values are expressed as the mean ± standard deviation (SD). The statistical analyses were completed with Student’s *t*-test or one-way analysis of variance (ANOVA) followed by Tukey’s *post hoc* test. Differences were considered significant at *p* < 0.05.

## Results

### RA Induced the Differentiation of SH-SY5Y Cells

**Figure 1:**
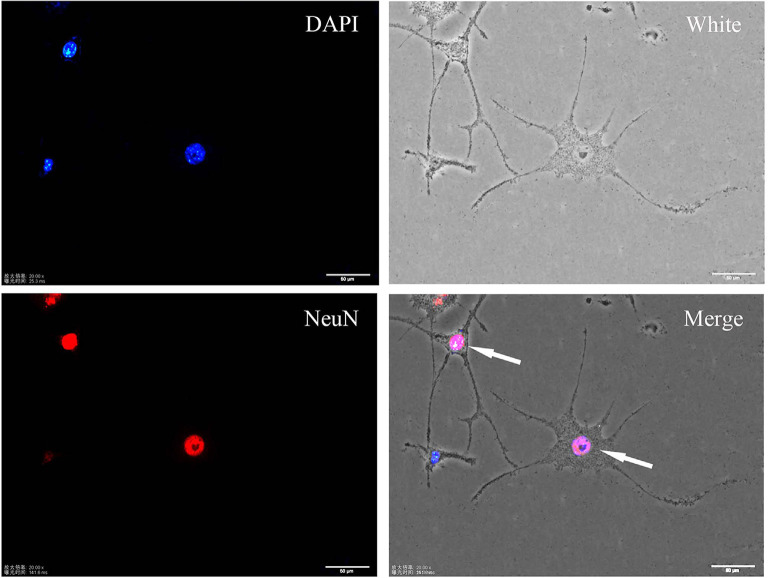
Immunofluorescence staining of the neuronal marker NeuN in differentiated SH-SY5Y cells. Differentiated cells show more protrusions and branches, and they are more neuron-like.

SH-SY5Y cells are widely utilized for *in vitro* studies aiming to determine the pathogenic mechanisms of neurodegenerative disorders (Pahrudin Arrozi et al., 2017). These cells are considered neuronal precursors. In this study, SH-SY5Y cells were induced to differentiate by RA. After treatment with 10 μM RA for 7 days, neuron-like changes in morphology, which were indicated by increased branches and neurite density, were observed in SH-SY5Y cells. Immunofluorescence staining showed that differentiated cells expressed the mature neuron marker NeuN (see Figure 1).

### The Monomer HF-488-Aβ_1–42_ Entered Cells and Caused a Decrease in the Expression of PSD-95

**Figure 2:**
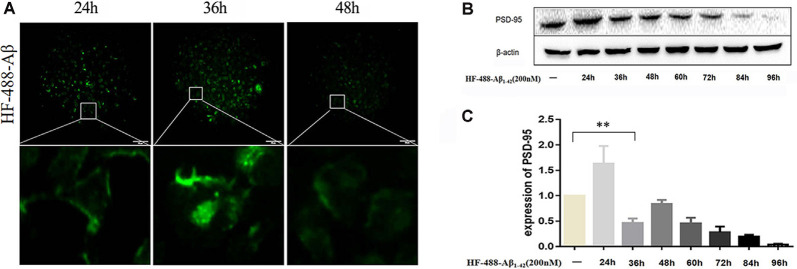
Intracellular HF-488-Aβ fluorescence intensity and the effect of Aβ on PSD-95 expression. **(A)** The fluorescence intensity of cells treated with 200 nM HF-488-Aβ for different amounts of time (24, 36, and 48 h). **(B)** Immunoblot analysis of PSD-95 from cells treated with 200 nM HF-488-Aβ for different amounts of time (24, 36, 48, 60, 72, 84, and 96 h). **(C)** Quantification of PSD-95 normalized to β-actin and expressed as a % of control shows the significant differences between SH-SY5Y cells and cells treated with HF-488-Aβ for 36 h. All values are presented as the mean ± standard deviation (SD) from three independent experiments. *n* = 5. **p < 0.01 vs. control.

After treating SH-SY5Y cells with green fluorescence-labeled HF-488-Aβ_1–42_, there was obvious fluorescence in the cells at 24 h, and the fluorescence intensity reached its maximum at 36 h before gradually decreasing (see Figure 2A). PSD-95 expression was increased at 24 h (*p* < 0.01), while it began to significantly decrease at 36 h (*p* < 0.01). After that, the expression is gradually decreased (in Figures 2B,C). Therefore, 200 nM HF-488-Aβ_1–42_ was used for 36 h in SH-SY5Y cells as an AD-related cellular model in which Aβ aggregates were not formed, but synaptic protein had changed.

### BG45 Increased Cell Viability, Increased the Expression of PSD-95 and Spinophilin, and Improved the Cytoskeleton

**Figure 3:**
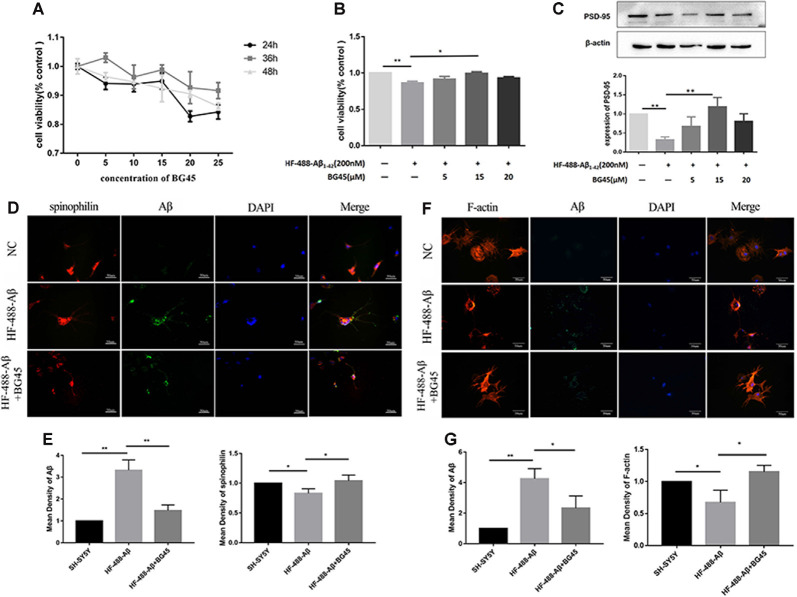
The effect of BG45 on cell viability, expression of PSD-95 and spinophilin, and the cytoskeleton. **(A)** The viability of cells treated with vehicle or BG45 (5, 10, 15, 20, and 25 μM) for 24, 36, and 48 h. **(B)** The cell viability of cells treated with vehicle, HF-488-Aβ (200 nM) or HF-488-Aβ (200 nM) + BG45 (5, 15, and 20 μM). **(C)** Immunoblot analysis of PSD-95 from cells treated with vehicle, HF-488-Aβ or HF-488-Aβ + BG45 (5, 15, and 20 μM) is shown as lanes. Quantification of PSD-95 is normalized to β-actin and is shown as a % of control, which reveals the significant differences between SH-SY5Y cells and HF-488-Aβ treated cells, as well as the differences between cells treated with HF-488-Aβ alone and cells treated similarly but with an additional 15 μM BG45. **(D)** Immunofluorescence staining analysis of Aβ (green) and spinophilin (red) in SH-SY5Y cells treated with HF-488-Aβ or HF-488-Aβ + BG45 (15 μM) for 36 h. **(E)** Quantitative Aβ and spinophilin analysis. **(F)** Immunofluorescence staining analysis of Aβ (green) and F-actin (red) in SH-SY5Y cells treated with HF-488-Aβ or HF-488-Aβ + BG45 (15 μM) for 36 h. **(G)** Quantitative Aβ and F-actin analysis. All values are presented as the mean ± SD from three independent experiments. *n* = 5. *p < 0.05 and **p < 0.01.

In this study, the optimal time and concentration for treatment with BG45 were screened with CCK-8 assays. The results showed that the activity of normal SH-SY5Y cells was inhibited with 20 μM BG45 treatment for 48 h. Therefore, 15 μM BG45 was used for 36 h in this experiment (see Figure 3A). Furthermore, the effect of BG45 on the viability of Aβ-induced cells was detected. The results showed that compared with the normal group, treatment with 200 nM HF-488-Aβ_1–42_ for 36 h significantly decreased cell viability (*p* < 0.01), while the cell viability following treatment with 15 μM BG45 was significantly increased (*p* < 0.05; in Figure 3B). Similarly, the expression of PSD-95 in the Aβ-treated group was significantly downregulated (*p* < 0.01), and the expression of PSD-95 in the group treated with 15 μM BG45 was significantly higher than it was in the Aβ-treated group (*p* < 0.01; Krukowski et al., 2018; see Figure 3C).

Dendritic spines are the primary sites for receiving information and cellular substrates related to synaptic plasticity. Loss of spines often results in defective synaptic transmission (Cummings et al., 2015). We examined the changes in spinophilin and Aβ in SH-SY5Y cells treated with HF-488-Aβ or in cells exposed to BG45 after treatment with HF-488-Aβ. The results showed that the amount of Aβ in the HF-488-Aβ group was significantly increased compared to the control (*p* < 0.01), and the expression of spinophilin was significantly decreased (*p* < 0.05). Further, 15 μM BG45 reduced the intracellular Aβ content compared to the Aβ group (*p* < 0.01) and increased spinophilin (*p* < 0.05; in Figures 3D,E). We also analyzed the expression of Filamentous actin (F-actin). F-actin is the major cytoskeletal protein in spines, and spine structure is regulated by remodeling of the actin cytoskeleton, including those spines that develop during stabilization of memories after learning. The results also showed that BG45 can reduce Aβ levels and improve the cytoskeleton. Compared with the normal control group, the number of cell processes in the Aβ group decreased, the protrusions were shorter, and the expression of F-actin was significantly decreased (*p* < 0.05). In the BG45 treatment group, the amount of intracellular Aβ was significantly decreased (*p* < 0.05), while the expression of F-actin was increased and was accompanied by an increase in protrusions over what was observed in the Aβ group (*p* < 0.05; see Figures 3F,G).

### The Monomer HF-488-β_1–40_ Induced a Decrease in the Expression of PSD-95 in Mice

**Figure 4:**
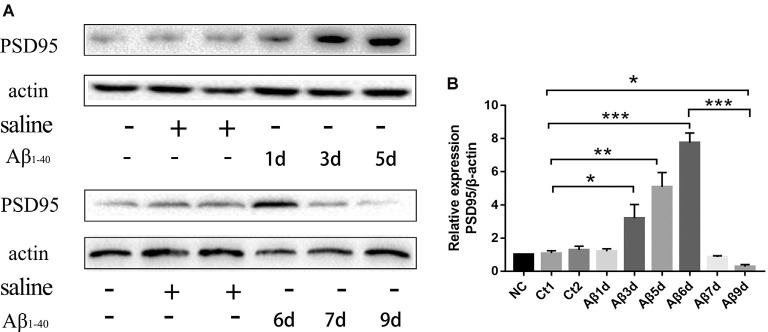
The expression of PSD-95 protein at different times after continuous injection of HF-488-Aβ_1–40_. **(A)** A representative band of the expression of PSD-95 in hippocampus from Wt mice, control mice with the vehicle and Aβ-treated mice for 1, 3, 5, 6, 7 and 9 days. **(B)** Quantification of PSD-95 is normalized to β-actin and is shown as a % of control. Compared with the control group, the expression of PSD-95 was temporarily increased for 6 days, but the expression was decreased significantly for 9 days. *n* = 5. *p < 0.05, **p < 0.01, ***p < 0.001.

PSD-95 was detected to find out how many days continuous injection of HF-488-Aβ_1–40_ can cause a decrease in synaptic protein, that is, may instantly cause synaptic damage. The results showed the expression of PSD-95 protein was not significantly changed for 1 day and was temporarily increased up to 6 days (*p* < 0.001), but decreased significantly for 9 days (*p* < 0.05) compared with the control group (see Figure 4). Therefore, 9 days of continuous injection was used as a mouse model of an early stage of Aβ-related neurodegenerative disease in the next experiments.

### HF-488-Aβ_1–40_ Appeared in the Hippocampus of Mice After Intravenous Injections

**Figure 5:**
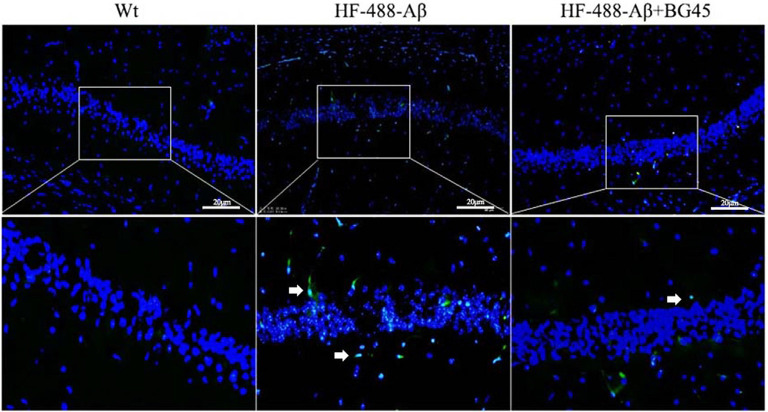
The fluorescence in the hippocampus after HF-488-Aβ_1–40_ intravenous injection. Merged image shows an overlay of HF-488-Aβ_1–40_ (green) and DAPI staining of the nucleus (blue) in the HF-488-Aβ and HF-488-Aβ + BG45 group. Arrows indicate the HF-488-Aβ_1–40_ in the hippocampus. *n* = 5. Scale bar corresponds to 50 μm.

Wang et al. (2017) confirmed that exogenous Aβ can enter the brain through the blood-brain barrier and cause AD-related pathological changes by injecting Aβ into the tail vein (Bu et al., 2017). At 9 days of continuous injection of green fluorescence-labeled HF-488-Aβ_1–40_, we found that the green fluorescence of HF-488-Aβ_1–40_ was present in the hippocampus in HF-488-Aβ group and HF-488-Aβ + BG45 group, while in the control group without HF-488-Aβ_1–40_ injection was negative (see Figure 5), which demonstrated that the HF-488-Aβ_1–40_ monomer entered the hippocampus through the blood-brain barrier.

### BG45 Decreased the Phosphorylation of Tau in HF-488-Aβ_1–40_-Treated Mice

**Figure 6:**
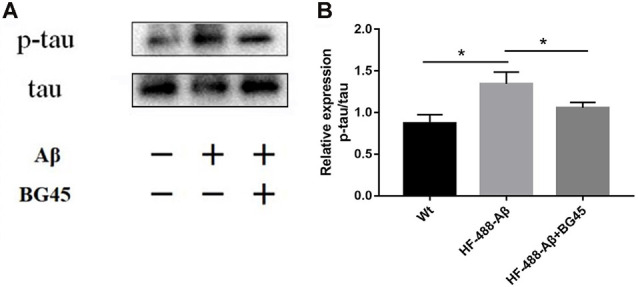
BG45 decreased the phosphorylation of tau. **(A)** Representative bands of the expression of p-tau/tau from control mice, HF-488-Aβ group and HF-488-Aβ + BG45 group. **(B)** Quantification of PSD-95 is normalized to tau and is shown as a % of control. Compared with the control group, exogenous Aβ treatment increased the phosphorylation level of tau protein. Compared with the HF-488-Aβ group, the phosphorylation level of tau protein in the HF-488-Aβ + BG45 group was lower. *n* = 5. *p < 0.05.

The hyperphosphorylation of tau protein plays an important role in the neurodegeneration and synaptic dysfunction of AD. The results showed that compared with the control group, the phosphorylation level of tau protein after exogenous Aβ treatment was significantly higher (*p* < 0.05), while BG45 reduced its expression (*p* < 0.05; see Figure 6).

### The Effect of BG45 on HDAC1, HDAC2 and the Expression of Synapse-Related Protein

**Figure 7:**
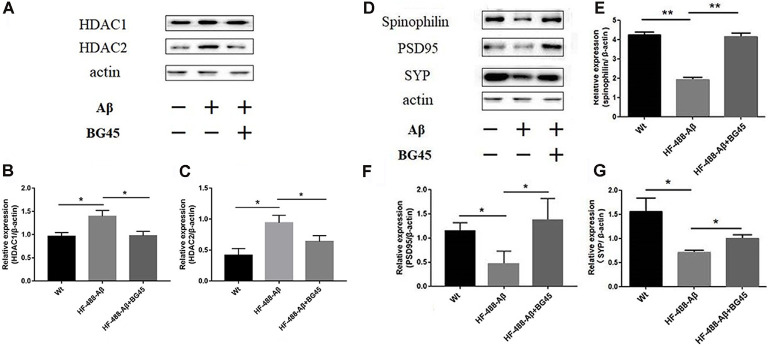
Effect of BG45 on the expression of HDAC1, HDAC2, synaptic-related proteins after the HF-488-Aβ_1–40_ treatment. **(A)** Representative bands of the expression of HDAC1 and HDAC2 from control mice, HF-488-Aβ group and HF-488-Aβ + BG45 group. **(B,C)** Quantification of HDAC1 and HDAC2 is normalized to β-actin and is shown as a % of control. The expression of HDAC1, 2 was increased in the HF-488-Aβ group. Compared with the HF-488-Aβ group, the expression was significantly decreased in the HF-488-Aβ + BG45 group. **(D)** Representative bands of the expression of synapse-related protein from the control group, HF-488-Aβ group, and HF-488-Aβ + BG45 group. **(E–G)** Quantification of spinophilin, PSD-95, and synaptophysin (SYP) is normalized to β-actin and is shown as a % of control. The expression of spinophilin, PSD-95, and SYP was significantly decreased in the HF-488-Aβ group compared with the control group, while was improved in the HF-488-Aβ + BG45 group. *n* = 5. *p < 0.05, **p < 0.01.

The levels of HDAC1 and HDAC2 were detected in the exogenous Aβ-treated mice with or without the administration of BG45. The results showed the levels of HDAC1 and HDAC2 in the exogenous Aβ treated group were significantly higher than those in the control group (*p* < 0.05, *p* < 0.05; see Figures 7A–C). In the BG45-treated group, the levels were slightly lower than those in the Aβ-treated group (*p* < 0.05, *p* < 0.05). Meanwhile, the effect of BG45 on the synapse-related protein was detected. Consistent with the results *in vitro*, the expression of spinophilin was significantly decreased by exogenous HF-488-Aβ_1–40_, while BG45 prevented the effect from the Aβ_1–40_ (*p* < 0.01). The expression of PSD-95 and SYP was also down-regulated by exogenous Aβ (*p* < 0.05, *p* < 0.05), whereas BG45 increased the expression of PSD-95 and SYP (*p* < 0.05, *p* < 0.05; see Figures 7D–G).

### BG45 Upregulated the Level of *GRIK2, SCN3B*, and *SYNPR* Gene and Protein

**Figure 8:**
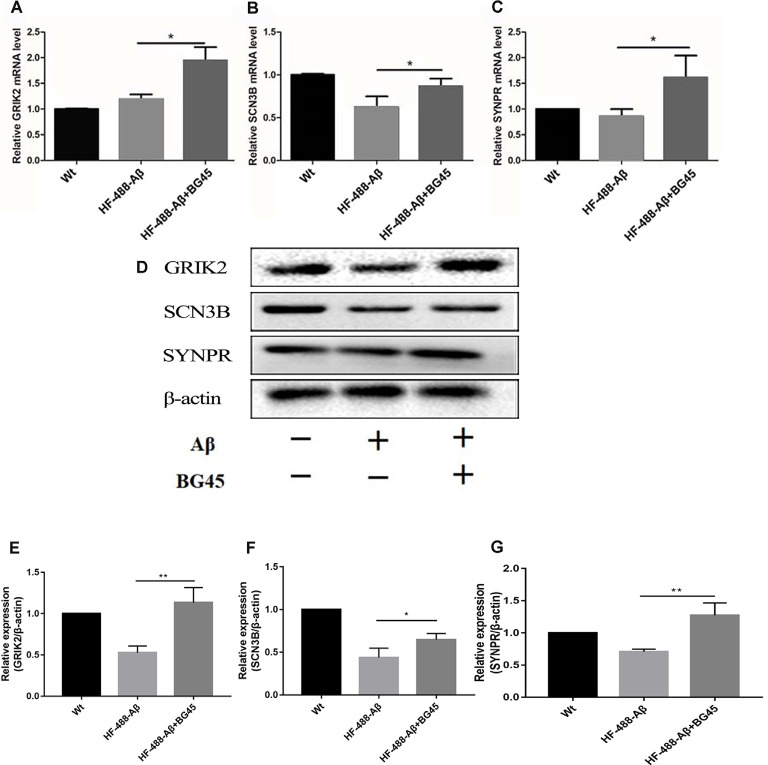
The mRNA and protein levels of synaptic-related genes in the hippocampus after HF-488-Aβ treatment. **(A)** the mRNA levels of GRIK2; **(B)** the mRNA levels of SCN3B; **(C)** the mRNA levels of SYNPR. **(D)** Representative bands of the expression of GRIK2, SCN3B, and SYNPR from control mice, HF-488-Aβ group, and HF-488-Aβ + BG45 group. **(E–G)** Quantification of GRIK2, SCN3B, and SYNPR is normalized to β-actin and is shown as a % of control. Compared with the HF-488-Aβ group, the mRNA levels and their proteins of synaptic-related genes GRIK2, SCN3B, and SYNPR were upregulated in the HF-488-Aβ + BG45 group. *n* = 5. *p < 0.05, **p < 0.01.

To investigate the potential mechanism of BG45 in increasing the expression of synaptic proteins, we also detected several synapse-related genes and proteins which are possibly involved in the role of BG45 (Yamakawa et al., 2017). The results showed that compared with the mice treated with only HF-488-Aβ, BG45 did improve the levels of *GRIK2, SCN3B*, and *SYNPR* gene (*p* < 0.05, *p* < 0.05, *p* < 0.05; see Figures 8A–C) and their corresponding proteins (*p* < 0.01, *p* < 0.05, *p* < 0.01; see Figures 8D–G).

## Discussion

In a recent study, it was found that in mouse models, early AD did not affect the encoding of memory but only prevented the mice from recalling existing memories. The researchers used optogenetic techniques to increase dendritic spines and brought previously “unreadable memories” back to the mice (Roy et al., 2016). Currently, optogenetic techniques may not be suitable for clinical treatment, so we tried to find a medicine that could specifically repair dendritic spines in the hippocampus or other brain regions. In this study, neuroblastoma SH-SY5Y cells treated with exogenous Aβ or intravenous injection of exogenous Aβ to mice were used as an AD-related model. We found that exogenous Aβ monomers caused the changes in synaptic proteins and cytoskeleton *in vitro* or *in vivo*, and we also found that the class I HDACI, BG45, improved synaptic damage.

In this study, the differentiated neuroblastoma cells SH-SY5Y were found to express the neurospecificity nuclear proteins NeuN. These cells showed some neuron-like features, such as protuberances and branches. Aβ is considered a key factor in the pathogenesis of AD, and the plaque-like deposit formed by Aβ aggregation is a significant pathological development in AD. Exogenous Aβ can induce cell synaptic damage and produce cytotoxicity. Some researchers treated cells with HF-488-Aβ_1–42_ and observed intracellular Aβ aggregation by fluorescence lifetime imaging microscopy (Bolognesi et al., 2013). The state of Aβ can affect its fluorescence effect. Fluorescence quenching occurred when Aβ aggregates form a precipitate (Winters et al., 2014). In this study, SH-SY5Y cells were treated with 200 nM HF-488-Aβ_1–42_, and the intracellular fluorescence intensity was observed to determine the degree of intracellular Aβ aggregation. The results demonstrated that the intracellular fluorescence intensity reached a maximum value at 36 h and then gradually weakened over time. It is speculated that Aβ aggregates have not formed at 36 h. On the other hand, changes in synapses are most closely related to cognitive dysfunction, and synaptic function disorders precede the prevalence of extensive neuronal degeneration (Gylys et al., 2007; Terry et al., 2010). PSD-95 is a postsynaptic marker protein that plays an important role in the development and function of neurons (Matt et al., 2019). The expression of PSD-95 protein in different periods was examined to elucidate the early synaptic damage in SH-SY5Y cells induced by HF-488-Aβ_1–42_. The results showed that the expression of PSD-95 was increased in comparison with the control group at 24 h after Aβ treatment. While the expression of PSD-95 was significantly decreased at 36 h, the expression gradually decreased. These results suggest that very low concentrations of Aβ may begin to have a beneficial effect on neurons (Garcia-Osta and Alberini, 2009), but if the Aβ monomers persist, then synaptic damage may have been caused when the monomers have not formed Aβ oligomers at 36 h. Therefore, SH-SY5Y cells treated with HF-488-Aβ_1–42_ for 36 h were used as an early AD-related cell model in subsequent experiments.

The cytoskeleton plays an important role in maintaining the structure and function of neurons, and it is associated with the occurrence of a variety of neurodegenerative diseases (Rudrabhatla, 2014). Moreover, Aβ, as a key protein molecule in the pathogenesis of AD, can induce abnormal stability of actin fibers (F-actin) in dendritic spines, thereby impairing synaptic plasticity (Rush et al., 2018). In this study, treatment with 200 nM HF-488-Aβ for 36 h reduced cell protrusions and decreased the expression of cytoskeleton F-actin, which demonstrated cytoskeletal damage. BG45, a class I HDACI, significantly alleviated the loss of cell viability caused by the addition of exogenous Aβ before forming aggregates; BG45 also increased PSD-95 and spinophilin expression and repaired cytoskeleton damage. Here, we deduced that BG45 may enhance the expression of synaptic proteins involved in the stabilization of the actin cytoskeleton in dendritic spines by specifically decreasing the levels of class I HDACs.

To further confirm the above conclusion, we investigated the neuroprotection of BG45 on animals injected with HF-488-Aβ_1–40_ through the tail vein to induce synaptic damage in brain tissue. There are several differences in the concentration of Aβ monomers between the central and peripheral pools. Aβ_1–42_, which is the most neurotoxic form of Aβ, is the dominant molecular species in the brain, whereas Aβ_1–40_ is the main form in the periphery (Wang et al., 2017). The researchers found that the human Aβ originated from APPsw/PS1dE9 transgenic AD mice could enter the brain of Wt mice by using a model of parabiosis between the transgenic AD mice and their wild-type littermates and caused Aβ deposition, over-phosphorylation of tau, cerebrovascular pathological changes and damage of LTP in hippocampal CA1 area in the Wt mice (Bu et al., 2017). It revealed that blood-derived Aβ entered the brain and induced Aβ-related pathologies and functional impairment of neurons. In this study, intravenous injection of Aβ_1–40_ monomers was used to establish a model to explore that Aβ_1–40_ entered the hippocampus from peripheral blood and caused early AD-related pathological changes.

Consistent with the results *in vitro*, we found that the treatment of exogenous Aβ stimulated the increase of PSD for a short period, and then the continuous injection of Aβ led to the decrease of PSD expression. This further suggests that Aβ may play an active protective role in the early stage of AD, which is consistent with previous studies that showed in the early stage, Aβ with a very low concentration (picomolar range) does not damage synapses, but has the function of protecting neurons and regulating memory consolidation (Garcia-Osta and Alberini, 2009; Giuffrida et al., 2009). But Soluble Aβ could affect the NFTs generation by controlling the cleavage and phosphorylation of tau (O’Brien and Wong, 2011). Because of the excessive Aβ in the organism, tau protein will induce hyperphosphorylation while contacting with kinase (Tiwari et al., 2019). This high level of phosphorylated tau protein will lead to the unstable depolymerization of tubulin to filaments, and further lead to insoluble NFTs, leading to neuronal damage (Marcus and Schachter, 2011). In this study, the phosphorylation of Tau was indeed increased when Aβ caused a decrease of PSD-95 on the 9th day of continuous injection. But BG45 reversed the negative effect of Aβ monomers from peripheral blood.

When we explored the correlation between HDACs and synapse-related proteins, we found that Aβ monomers from peripheral blood upregulated the expression of HDAC1 and HDAC2, accompanied by a decrease in the expression of synapse-related proteins in the hippocampus. However, BG45 can improve the expression of pre- and post-synaptic proteins (SYP, PSD-95, and spinophilin) which are highly associated with the formation of the synapse. HDACs may play a mediating role in the dendritic spine injury induced by Aβ oligomer (Ishizuka et al., 2014). It has been pointed out that in class I HDACs, HDAC2 is enriched in promoters of genes related to neuronal activity regulation and synaptic plasticity (Yamakawa et al., 2017), indicating that HDAC2 plays an important role in regulating gene expression related to neuronal function. There may be a negative regulatory relationship between HDAC2 and synaptic plasticity (Ji-Song et al., 2015). It is very important to inhibit HDACs, especially HDAC2, to recover the memory impairment caused by aging or nervous system diseases (Singh and Thakur, 2018), which may be due to remodeling chromatin and enhancing gene expression.

In the process of gene transcription, HDAC is not directly bound to DNA but is recruited to the required gene through DNA binding protein-like *Sp3*, resulting in dynamic acetylation of histone and the transcription factors (Sun et al., 2002). HDAC2-Sp3 complex localizes to the promoters of synaptic-related genes where it deacetylates histone substrates. The complex further co-regulates synaptic genes and inhibits the synaptic plasticity of neurons. In CK-p25 mice and AD patients, synaptic genes such as *Dlgap1, Gabbr2, Scn3b, and Synpr* were downregulated (Liang et al., 2008; Gjoneska et al., 2015). The decrease in HDAC2 binding due to the knockdown of Sp3 is accompanied by an increase in histone acetylation at the promoters of multiple genes including *GRIK2, Dlgap1, and Gabbr2* (Yamakawa et al., 2017). In our study, the mRNA levels of *GRIK2*, *SCN3B, and SYNPR* were upregulated by BG45, which suggests that class I HDACs inhibitor may regulate the expression of these synaptic-related genes by decreasing the expression of HDAC2, further improve the expression of synaptic proteins and play a role in protecting synapses from damage.

In conclusion, as the HDAC class I-specific inhibitor, BG45 could significantly alleviate the down-regulation of PSD-95 protein and repair the damage of cytoskeleton induced by exogenous Aβ in SH-SY5Y cells. *In vivo*, BG45 might upregulate the level of synaptic-related genes through specifically inhibiting class I HDACs (HDAC1 and 2), further increase the expression of spinophilin, upregulate pre- and post-synaptic protein expression, thereby possibly improving synaptic plasticity in the early stage of the AD animal model. However, further studies are required to examine the effect of BG45 on functional synaptic plasticity in animal models.

## Data Availability Statement

The original contributions presented in the study are included in the article, further inquiries can be directed to the corresponding author/s.

## Ethics Statement

The animal study was reviewed and approved by Institutional Animal Care and Use Committee of the Dalian Medical University in Dalian, China.

## Author Contributions

We would like to thank LC for her help during the research. YG and CW: collecting the data. CZ and LK: providing key technical support. All authors contributed to the article and approved the submitted version.

## Conflict of Interest

The authors declare that the research was conducted in the absence of any commercial or financial relationships that could be construed as a potential conflict of interest.
